# Surveillance for Soil-Transmitted Helminths in High-Risk County, Mississippi, USA

**DOI:** 10.3201/eid2912.230709

**Published:** 2023-12

**Authors:** Richard S. Bradbury, Lora Martin, Lacy Malloch, Maygan Martin, John M. Williams, Kayla Patterson, Cameron Sanders, Gurbaksh Singh, Irene Arguello, Eduardo Rodriguez, Paul Byers, Lisa Haynie, Yvonne Qvarnstrom, Charlotte V. Hobbs

**Affiliations:** Federation University, Melbourne, Victoria, Australia (R.S. Bradbury);; University of Mississippi Medical Center, Jackson, Mississippi, USA (L. Martin, L. Malloch, M. Martin, J.M. Williams, K. Patterson, C. Sanders, G. Singh, I. Arguello, L. Haynie, C. Hobbs);; Oak Ridge Institute for Science and Education, Oak Ridge, Tennessee, USA (E. Rodriguez);; Centers for Disease Control and Prevention, Atlanta, Georgia, USA (E. Rodriguez, Y. Qvarnstrom);; Mississippi State Department of Health, Jackson (P. Byers)

**Keywords:** soil-transmitted helminths, hookworm, parasites, *Necator americanus*, *Strongyloides*, *Enterobius*, pediatric, Mississippi, United States

## Abstract

Recent reports of hookworm infection in Alabama, USA, has prompted surveillance in Mississippi, given the states’ similar environmental conditions. We collected stool specimens from 277 children in Rankin County, Mississippi. Kato–Katz microscopic smear, agar plate culture, and quantitative PCR indicated no soil-transmitted helminths. Nevertheless, further surveillance in other high-risk Mississippi counties is warranted.

Soil-transmitted helminths (STHs) are endemic in resource-limited settings worldwide. Because of its subtropical climate and socioeconomic factors, the southeastern United States historically has been at elevated risk for STH diseases. Because of improved sanitation and economic development, hookworm and other STHs were assumed to be eliminated from the US South (*1*). Mississippi has many areas with poor sanitation, but STHs have not been reportable since 1984 (*2*). Locally, expertise in clinical microscopic methods for STH diagnosis is lacking. At the University of Mississippi Medical Center (Jackson, MS, USA), stool samples are sent to the Mayo Clinic (Rochester, MN, USA), for routine ova and parasite examinations.

A recent report of suspected locally acquired cases of strongyloidiasis and hookworm in Alabama (*3*) initiated a parallel STH surveillance program in historically high-prevalence counties of Mississippi. After review of regions with high-risk criteria, including those with the soil type most conducive to human hookworm transmission (i.e., loamy soil) (*4*), review of Rockefeller Sanitary Commission data of areas with historically high prevalence, and review of sanitation data with the Mississippi Department of Health, we identified Rankin County as high risk. During 1910–1914, the Rockefeller Sanitary Commission found a very high prevalence of hookworm infection (42.1%) in Rankin County (*5*). The last formal surveillance study in Mississippi (1932–1933) found the prevalence in this county had decreased to 3.8% (*5*).

## The Study

We conducted a cross-sectional study in which we recruited parents and guardians of children in Rankin County at health fair and community events (February 2020–September 2021) and asked participants to submit 3 stool specimens per participating child (Appendix). Participants submitted specimens directly to designated clinics or drop sites, where study personnel collected the specimens. Because of the connection between STH and poverty (*3*), we targeted areas within the county that had higher deprivation indices (https://www.neighborhoodatlas.medicine.wisc.edu).

We preserved ≈250-mg aliquots of fresh stool specimens in 70% ethanol at room temperature. For DNA extraction, we washed the specimens once in phosphate-buffered saline, followed by freezing at –80°C for 30 min, snap-thawing at 100°C for 10 min, then bead beating for 1 min with zirconium beads (Benchmark, https://www.benchmarkscientific.com) instead of the kit-supplied beads. Otherwise, we performed DNA extraction according to the manufacturer’s instructions. We processed the first 128 specimens by using the SurePrep Soil DNA Isolation Kit (Fisher Scientific, https://www.fishersci.com). This kit was discontinued by the manufacturer during the study, so we validated and processed the remaining 660 specimens by using the PowerFecal ProDNA kit (QIAGEN, https://www.qiagen.com) (*6*). We immediately stored DNA extracts at –80°C and sent them to the Division of Parasitic Diseases and Malaria, Center for Global Health, at the Centers for Disease Control and Prevention for quantitative PCR (qPCR) analysis.

Where specimen volume allowed, we performed microscopic analysis by using saturated sodium nitrate (NaNO_3_) centrifugal flotation as previously described but with a 500 × *g* (instead of 3,000 × *g*) centrifugation step (*7*). We performed Kato–Katz microscopy as previously described (*8*). We prepared Koga agar plate cultures (APCs) and inoculated as previously described (*9*). We sealed the plates with parafilm, incubated them at 28°C, and checked for larval tracks on days 3 and 5.

We quality-control tested DNA extracts for PCR inhibitors by using a human cytochrome B qPCR (*10*). We tested DNA samples without inhibition by using multiparallel qPCR specific for *Necator americanus*, *Ancylostoma duodenale*, *Trichuris trichiura*, *Strongyloides stercoralis* (*11*), and *Ascaris lumbricoides* (*12*). We considered a cycle threshold value <40 to be positive. We incorporated positive (genomic DNA from worms) and negative (water and DNA extracted from STH-free stool specimens) controls into each qPCR run.

We collected data regarding risk factors for STH by using case report forms from parents or guardians representing 354 children 2–18 years of age at the time of enrollment. The median age of children enrolled was 8 years (interquartile range 5.0–11.5 years); 55.4% were boys and 44.6% girls; 78.2% were White and 16.9% Black (US Census data for Rankin County [https://www.census.gov/quickfacts/rankincountymississippi] indicate the population is 74.5% White and 22.5% Black) (Table 1). Most (94.6%) reported non-Hispanic ethnicity, although US Census data indicate 72.2% are non-Hispanic in this county (Table 1). According to survey responses, 12.5% had traveled outside the United States in the previous 5 years, 7% had prior treatment for an intestinal parasite (80% had treatment for *Enterobius vermicularis* pinworm), most (89.7%) children had some contact with soil, and all had a flushable toilet (Table 2; Appendix Table [data for children for whom stool specimens were received]).

**Table 1:** Demographic characteristics of 354 school-age children enrolled in a study of soil-transmitted helminth infection, Rankin County, Mississippi, USA, February 2020–September 2021*

Characteristic	No. (%)	
Sex		
M	196 (55.4)	
F	158 (44.6)	
Total	354 (100)	
Ethnicity†		
Hispanic	15 (4.3)	
Not Hispanic	332 (94.6)	
Prefer not to answer	3 (0.8)	
Unknown	1 (0.3)	
Total	351 (100)	
Race		
White	277 (78.2)	
Black or African American	60 (16.9)	
Asian	2 (0.6)	
AI/AN	0	
NHOPI	0	
White and Black or African American	10 (2.8)	
White, Asian, and AI/AN	2 (0.6)	
White and AI/AN	1 (0.3)	
White, AI/AN, and NHOPI	1 (0.3)	
Unknown	0	
Prefer not to answer	1 (0.3)	
Total	354 (100)	

*Median age of children enrolled was 8 years (interquartile range 5.0–11.5 years). AI/AN, American Indian or Alaska Native; NHOPI, Native Hawaiian or Other Pacific Islander. †Records missing for 3 children.

**Table 2:** Selected risk factors for soil-transmitted helminth infection among 354 school-age children enrolled in study of soil-transmitted helminth infection, Rankin County, Mississippi, USA, February 2020–September 2021

Question and answer	No. (%)
Has your child travelled outside the United States in the past 5 years?	
Yes	44 (12.5)
No	308 (87.5)
Total	352* (100)
Has your child been treated for intestinal parasites?	
Yes	24 (7)
No	322 (91)
Not sure	7 (2)
Total	353* (100)
If yes to treatment for an intestinal parasite above, which one?	
Hookworm	1 (4)
Roundworm	0
Whipworm	0
Pinworm	20 (80)
Not sure	4 (16)
Total	25 (100)
Has your child played outside with bare hands or bare feet (has there been contact with soil) in the past 3 years?	
Never	34 (9.7)
Sometimes/less than once a month	61 (17.3)
Often/at least monthly	104 (29.5)
All the time	151 (42.9)
Not sure	2 (0.6)
Total	352* (100)
If yes to contact with soil as above, how often?	
Daily	31 (12.1)
Weekly	81 (31.8)
Monthly	104 (40.8)
Yearly	39 (15.3)
Total	255 (100)
What type of toilet is in the home where your child lives?	
Flushable toilet†	350* (100)

*Data were missing for some of the 354 children. †Questionnaire response options were flushable toilet, outdoor toilet, or prefer not to answer.

We received 784 stool specimens from 277 of the 354 survey respondents, representing 129 households (Figure). Three specimens taken over 3 days were submitted by 245 participants, 15 submitted only 2 specimens, and 17 submitted a single specimen. Laboratory processing was performed within 72 hours of specimen collection for 98% of specimens received. Sufficient specimen was present to perform NaNO_3_ flotation microscopy on 731 specimens. We performed Kato–Katz microscopic analysis on 730 specimens and conducted APC on 728 specimens. All NaNO_3_ flotations, Kato–Katz microscopy, and APC tests showed no STH. Two participants (0.6%) were positive in 1 of 3 specimens each for *Enterobius vermicularis* eggs by NaNO_3_ flotation only.

**Figure:**
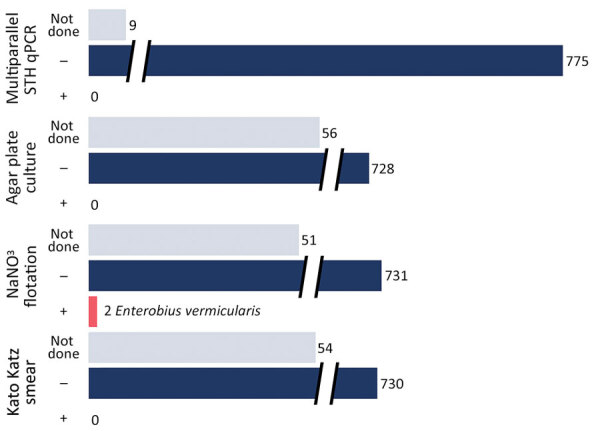
Results of microscopic analyses using Kato–Katz smear, saturated NaNO_3_ flotation, and agar plate culture for *Strongyloides* and hookworm larvae, in addition to multiparallel qPCR results, for 784 stool specimens from 277 school-age children representing 129 households in a surveillance study for STH infection, Rankin County, Mississippi, USA, February 2020–September 2021. Multiparallel qPCR targeted *Necator americanus*, *Ancylostoma duodenale*, *Trichuris trichiura*, *Strongyloides stercoralis,* and *Ascaris lumbricoides*. *Enterobius vermicularis* eggs only were recovered in the 2 positive NaNO_3_ flotation results. PCR could not be performed for 9 specimens from 9 children, but all were negative by Kato–Katz smear and saturated NaNO_3_ flotation, agar plate culture for *Strongyloides*, and hookworm larvae. Of those, 8/9 were from children who submitted 3 specimens total each and whose other specimens tested negative by PCR and 1 child submitted only 1 specimen. NaNO_3_, sodium nitrate; ND, not done; qPCR, quantitative PCR; STH, soil-transmitted helminth; –, negative; +, positive.

We extracted DNA from all specimens and subjected them to qPCR analysis. Negative results in a DNA quality-control assay excluded 9 specimens (1.15%) from further qPCR analysis. We subjected the remaining 775 DNA extracts to multiparallel STH qPCR, all of which were negative. Of note, the 9 excluded specimens were all negative by NaNO_3_, Kato–Katz microscopic analyses, and APC.

## Conclusions

Our findings suggest the absence of STH infections among the children surveyed in Rankin County, Mississippi. We suspect that our survey results may not have captured sanitation and hygiene data: a limitation of our survey design was that it did not enquire about the endpoint of flush toilets, so any household effluent released nearby through straight pipes was possibly not detected by our survey tool. Furthermore, a stigma associated with having substandard sanitation or fear of ramifications for the need to install appropriate sanitation may have limited the veracity of some responses. However, our laboratory data are consistent with our prior surveillance in the Mississippi Delta (*10*) and work with postdiagnostic specimens in Mississippi (*2*), which demonstrated no human hookworm, *Ascaris*, or *Trichuris* spp. infections by microscopic analysis or qPCR. Rare *S. stercoralis* infections were detected previously in Mississippi residents by serologic analysis, but whether those infections were autochthonously acquired, travel-acquired, or chronic or persistent infections many decades after exposure is unclear (*2*). We have observed sporadic cases of *Ascaris* hookworm infections in Mississippi (C. Hobbs, unpub. data), usually in association with pig farming, and those cases may therefore represent zoonotic acquisition of *A. lumbricoides* pig genotype. Both hookworm and *S. stercoralis* worms are common in children in STH-endemic areas globally but increase in prevalence with age (*13*,*14*). Further surveillance of adults might identify rare STH infections, particularly persistent cases of strongyloidiasis. Even though 12.5% of respondents in our investigation had traveled outside of the United States, none harbored travel-acquired helminthic infections.

The prevalence of enterobiasis was much lower in our study compared with the most recent surveillance from the United States, which reported prevalence of 11.6%–38.9% in southern California elementary schools in the early 1980s (*15*). Our results may represent actual lowered prevalence or may be attributable to the poor sensitivity of NaNO_3_ flotation for *E. vermicularis* detection.

Further study is needed in several other counties that have had historically high levels of hookworm infection. However, considered in the context of other recent surveillance studies (*2*,*10*), it is becoming increasingly likely that continued transmission of STH is not occurring in this high-risk county in Mississippi.

Appendix Additional information about surveillance for soil-transmitted helminths in a high-risk county, Mississippi, USA.
