# Supplementary material for: Surveillance for Soil-Transmitted Helminths in High-Risk County, Mississippi, USA
Source: Emerg Infect Dis. 2023 Dec;29(12):2533–7. doi: 10.3201/eid2912.230709 (PMC10683803; doi:10.3201/eid2912.230709)
Supplement: Appendix — Additional information about surveillance for soil-transmitted helminths in a high-risk county, Mississippi, USA. [file 23-0709-Techapp-s1.pdf]

Article DOI: https://doi.org/10.3201/eid2912.230709

EID cannot ensure accessibility for supplementary materials supplied by authors. Readers who have difficulty accessing supplementary content should contact the authors for assistance.

Surveillance for Soil-Transmitted Helminths in High-Risk County, Mississippi, USA

Appendix

Additional Methods

Inclusion criteria included that informed consent be given by parent/guardian, children be aged 2 to 18 years of age at enrollment, and be resident of Rankin county. Informed consent was obtained from parent or guardian and for all participants, and assent was obtained for those aged 9 or older, consistent with University of Mississippi Medical Center Institutional Review Board requirements. Parents/guardians were asked to complete a questionnaire (demographics, clinical and travel history, and information on potential risk factors for soil-transmitted helminth (STH) infections and to collect three stool samples on non-consecutive days from each participating child. For sample size, calculations were based on an estimate of 25% prevalence, with a 95% confidence interval and a difference worth detecting +/− 5% (d = 0.05), resulting in a required sample size of ≅246 subjects. Since there are not recent reliable hookworm prevalence data for Rankin county, these numbers were assumed based on MSDH reported parasitic disease statistics from 1980–1984 (Bruce Brackin, pers. comm.).

Appendix Table. Demographics of 271 school-aged children resident in Rankin county, Mississippi enrolled in this study who also contributed at least one stool sample*

Characteristic	Answer	Number (Responses)	Percent (%)
Sex	Male	153	56.5
	Female	118	43.5
	Total	271	100
Ethnicity	Hispanic	7	2.6
	Not Hispanic	260	95.9
	Prefer not to answer	0	0.0
	Unknown	1	0.4
	Total*	271	100
Race	White	216	79.7
	Black/ African American	41	15.1
	Asian	2	0.7
	American Indian/ Alaska Native	0	0.0
	Native Hawaiian/Pacific Islander	0	0.0
	White/ Black/ African American	8	3.0

Characteristic	Answer	Number (Responses)	Percent (%)
	White/Asian/American Indian/Alaska Native	2	0.7
	White/American Indian/Alaska Native	1	0.4
	White/ American Indian/ Native Hawaiian Pacific Islander	1	0.4
	Unknown	0	0.0
	Prefer not to answer	0	0.0
	Total	271	100.0

*Six subjects submitted stools but did not complete surveys and are therefore not included in this table. Median age in years (IQR) 8 (5.0-11.5).
